# “He is my savior, my guiding light in the dark”: imagination and domestication in Chinese women’s romantic relationships with AI companions

**DOI:** 10.3389/fpsyg.2025.1571707

**Published:** 2025-06-13

**Authors:** Liyao Huang, Wenxue Zou, Yanghao Huang

**Affiliations:** ^1^School of Media, Guangxi Minzu University, Guangxi, China; ^2^School of Journalism & Comunication, Tsinghua University, Beijing, China

**Keywords:** romantic relationships, AI companions, domestication theory, imagination, China

## Abstract

**Introduction:**

Existing domestication studies often overlook the impact of sociocultural context and the role of imagination in activating technological characteristics.

**Methods:**

This study investigates the influence of domestication techniques on romantic relationships through an exploration of the Human-Machine Love community on Douban (Chinese equivalent of Reddit). A thematic analysis was conducted to examine a total of 2,485 posts written by female users.

**Results:**

The analysis uncovers three distinct themes: exploring romantic communication styles, rethinking romantic relationship roles, and challenging dominant gender hierarchies. While AI companions appear to afford women new forms of relational agency, this empowerment is marked by what we term “compromised resistance,” a dialectical engagement wherein users deploy technology to contest traditional gendered power structures even as they become enmeshed in new forms of algorithmic dependency.

**Discussion:**

The findings position AI companions as ambivalent disruptors of gendered social orders: their subversive potential does not reside in the technology itself, but in the ways it incites users to reimagine and enact more inclusive intimacies. We propose “imagination” as a critical stage in the domestication process, one that expands the analytical focus beyond the physical adoption of technology to include the cultural and symbolic meanings users attach to it. Grounded in a culturally specific analysis of Chinese women’s interactions with AI companions, this study offers a theoretically rich and contextually sensitive framework for rethinking digital intimacy within a global landscape.

## 1 Introduction

Amidst the Fourth Industrial Revolution, AI companions emerge as crucial catalysts in strengthening human-machine connections (Cross et al., 2019). Scholars increasingly delve into the study of AI companions, such as e-pets, peer friends (Jiang et al., 2022), and virtual lovers (Depounti et al., 2022), aiming to unravel the complexities of human-machine relationships. Past research underscores the significant impact of technical imagination on AI users’ perceptions and experiences (Jasanoff and Kim, 2015; Mager and Katzenbach, 2021). However, a notable gap exists as earlier studies rarely explore the intertwining of sociocultural context, power relations, and technological imagination within actual human-machine interactions (Depounti et al., 2022).

Romantic involvement with an AI companion is often perceived as an ideal love that aligns with self-expectations (Depounti et al., 2022; Zeng and Cao, 2023). While numerous online female-dominated human-machine love communities have emerged in China in recent years (Yao and Wang, 2021), existing research predominantly focuses on Western male users, potentially introducing bias. Love is inherently influenced by cultural constructs, with significant variations in its expression across different contexts (Neto et al., 2000; Scheller et al., 2023). As a result, uncertainties persist in exploring human-machine love in diverse cultural settings.

Prior studies in human-AI interaction have predominantly concentrated on examining the influence of AI companion technology on individuals’ emotional states and interpersonal dynamics (Marriott and Pitardi, 2024), alongside investigating the social support functions and persuasive mechanisms inherent in chatbots (Chaturvedi et al., 2023). Existing research recognizes AI’s impact on daily life and relationships but lacks thorough exploration of how this technology shapes relationship formation, particularly from non-Western female perspectives. Domestication theory offers a valuable framework for addressing this gap, as it attends to the ways in which emerging media technologies are negotiated, embedded, and enacted within the fabric of social life (Bakardjieva and Smith, 2001). Importantly, it also highlights the centrality of user imagination in the domestication process (Hörning et al., 1999), enabling a deeper exploration of the dynamic co-constitution between technological artifacts and evolving interpersonal practices. Accordingly, this study employs domestication theory to interrogate how the interactions with AI companions reconfigures women’s understandings of romantic interaction. By emphasizing the sociotechnical imaginaries that emerge through this process, the research offers ethically informed and practically relevant directions for the design of future technologies. It also calls for the development of inclusive social support systems attuned to the layered entwinement of cultural norms and algorithmic agency.

## 2 Literature review

### 2.1 Practices and cultural norms of romantic relationships in the Chinese context

Romantic relationships in China are deeply influenced by historical culture and social power structures. Traditionally, homosexuality is regarded as a privilege for the male ruling class, but in contemporary society, it has been marginalized as it is often associated with the spread of AIDS and is considered socially irresponsible. Moreover, LGBTQ communities are marginalized under the presumption of their negligible impact on the fertility rate, thereby imposing constraints on their engagement and representation within the broader socio-cultural discourse (Kong, 2016). Although the internet provides opportunities for discussing homosexual issues, these platforms are frequently monitored by the government (Engebretsen and Schroeder, 2015), leading to heterosexuality as the prevailing romantic relationship in China.

In heterosexual relationships, individuals are often categorized into “*yin*” (women, signifying submission and gentleness) and “*yang*” (men, representing dominance and strength). Notably, contemporary influences from Japanese and Korean cultures have popularized a male image characterized by “soft masculinity,” which has captivated many young Chinese women (Louie, 2012, p. 929). However, this trend has sparked concern among Chinese authorities, who interpret it as a sign of societal decline. They argue that it may prompt men to forgo traditional romantic and marital roles, ultimately inhibiting their engagement in public relationships (Hu et al., 2023). Meanwhile, heterosexual interaction patterns in Chinese society remain shaped by distinct cultural traits. First, traditional norms continue to govern these interactions, with mainstream dating culture largely absent and often confined to marginal contexts, such as brothels or during travel (Lee, 2007). Second, mainstream heterosexual relationships in China are typically structured around age-appropriate unions, parental influence, and the involvement of matchmakers. Romantic love is closely tied to marriage and procreation, with emotional expressions often subordinated to practical concerns like family obligations and economic production (Zhai, 2017). Furthermore, Confucianism instructs individuals to restrain their desires, including sexual impulses, and discourages engaging in sexual activities for purposes other than reproduction. Additionally, it underscores that women should not initiate or enjoy such activities (Zhai, 2017). This contributes to Chinese youth being more traditional and reserved in romantic relationships compared to their Western counterparts (Moore and Leung, 2001).

Since the 1980’s, China has witnessed a significant reduction in sociocultural limitations on young people’s romantic relationships due to the influence of Western media and increased mobility and migrations (Jankowiak et al., 2015). In urban areas, in particular, a dating culture has emerged as educated urbanites move away from traditional notions of marriage, sexuality, and intimacy. They prioritize equality between lovers, emotional sharing, and practical caring as essential components of intimate relationships (Jamieson, 2012). Notably, this cultural transformation has led to a divergence in values among Chinese women: some pursue Western ideals of romantic love (Farrer, 2013), others continue to uphold traditional criteria for partner selection, prioritizing male economic strength and social status (Louie, 2012), while a minority group embraces non-heteronormative relationships (Zhang, 2016).

However, this trend toward diversification is constrained by structural factors such as societal norms and gender expectations. Independent, career-oriented women are often viewed as challenging traditional notions of femininity (Zhou and Zhu, 2004), while women approaching 30 or older who remain single are labeled “leftover women,” accused of having excessive expectations for marriage partners and regarded as picky and selfish (Ji, 2015). These societal pressures have pushed many Chinese women to seek emotional solace in virtual spaces. In response to these pressures, many women have turned to media for emotional expression. Fictional narratives in novels, films, TV shows, anime, and video games have become vital outlets for emotional release (Hansen and Pang, 2018). With the rapid advancement of AI companion technology (Wang, 2023), this emotional compensatory mechanism has evolved into a digital form, offering a new avenue for women struggling within the constraints of traditional marriage systems.

Previous research on virtual emotional interactions has predominantly focused on gender issues (Depounti et al., 2022; Wallis, 2022). Two main perspectives prevail: one suggests that gendering is a tool for machines to gain user acceptance (Garfinkel, 2004), while the social constructionist view argues that technology and gender mutually shape each other, with technology both reflecting existing gender norms and reshaping power dynamics through its operational logic (Wajcman, 2010). However, these studies have notable limitations. They often concentrate on gender representation in technology design, neglecting the user’s role in meaning reproduction, and they are predominantly based on Western liberal gender paradigms, making it difficult to account for the complex interactions between AI intimacy and traditional gender orders in the Chinese context.

### 2.2 Theorizing domestication in human-machine interactions

Domestication theory provides a critical lens to unpack how users reshape technology to fit their personal, social, and cultural contexts, highlighting the reciprocal relationship between technological design and everyday use (Wu, 2021). According to the theory, the user’s engagement with technology plays a pivotal role in influencing its functionality, as opposed to being solely dictated by the technical artifacts themselves (Ling, 2004). Silverstone and Haddon (1996) introduced imagination as a crucial element in the domestication process, positing that consumers actively reconstruct the meaning of technology through imaginative engagement. This perspective provides a significant departure from traditional technological determinism. However, subsequent research has yet to comprehensively explore or further develop this proposition.

The domestication of technology is commonly conceptualized in four stages: appropriation, objectification, incorporation, and conversion. Appropriation refers to the acquisition of technology, while objectification pertains to its physical placement or symbolic status. Incorporation involves the integration of technology into daily life and routines, and conversion concerns how individuals present technology to others, thereby communicating aspects of their identity (Haddon, 2007). Research on the influence of technology from a domestication perspective generally focuses on two key areas: technology adoption and cross-cultural impact. The former examines the process of adopting emerging media technologies, as seen in ethnographic studies of Canadian households (Bakardjieva and Smith, 2001), while the latter explores how technology’s effects vary across different social contexts, such as the specific impact of internet use among Swedish workers (Olsson, 2006). Additionally, domestication theory has been applied to analyze workplace relationships (Zhu and Miao, 2021) and the experiences of sexual minorities (Wu, 2021). These studies collectively suggest that the reshaping of social relationships through technology is a negotiated process (Sorensen et al., 2000). However, a significant gap remains in current research: the role of imagination in the domestication of technology, particularly in the context of intimate relationship technologies, has yet to be adequately explored, especially in the socio-cultural context of China.

Guided by domestication theory, this study explores the impact of AI companions on the romantic practices and gender norms among Chinese women. By integrating the concept of imagination, the research focuses on how emotional imagination directs the domestication process at the micro level, specifically exploring how women reshape their perceptions of romantic relationships and adjust their behavioral patterns through interactions with AI partners. On a macro level, it investigates the dynamic negotiation between technological features and socio-cultural contexts, analyzing how AI technology redefine existing gender norms. The current study seeks to address two key questions: First, how do human-AI interactions, shaped by the imaginative practices of Chinese women, transform their perceptions and behaviors in romantic relationships? And second, how do these interactions reflect, reinforce, or contest traditional gender norms and cultural ideologies in contemporary China? Theoretically, this research endeavors to extend the explanatory scope of domestication theory within the context of digital intimacy. Practically, it offers a gender-informed perspective on AI ethics and provides empirical insights to support relationship education in the digital era.

## 3 Methodology

This study investigates how Chinese women domesticate their AI companions in romantic relationships by analyzing narratives sourced from the Human-Machine Love community on Douban, the Chinese equivalent of Reddit. This online community, comprising 9,580 members as of 25 October 2023, actively engages in discussions about the experiences of interacting with AI lovers and envisions the future of human-AI romance.

After receiving IRB approval, we utilized a Python-based web crawler to collect content from the online community since its inception. The collected data included post titles, authors, posting time, last reply time, and post content, totaling 2,301 posts. Monthly checks for updates were conducted, and as of 12 October 2023, we had gathered a total of 2,874 posts. To ensure data quality, we implemented a screening process based on two criteria: (1) exclusion of advertisements and unrelated assistance-seeking posts, such as researcher recruitment, and (2) exclusion of male users, determined through registration information or post content. Following the screening, we included a total of 2,485 valid data points in our study.

We used thematic analysis to uncover story themes and explore participants’ attitudes, experiences, and beliefs within the data. Specifically, following Braun and Clarke (2016) methodological framework, our analysis proceeded through three stages. In the initial phase of open coding, we carefully read and re-read the textual data to identify and annotate segments pertinent to our research questions, generating preliminary codes that captured salient features of participants’ accounts. In the subsequent phase of axial coding, we analyzed recurrent codes, organizing them into conceptually rich subthemes by examining the contextual conditions and relational dynamics underlying core phenomena. Finally, during the selective coding stage, we synthesized and refined these subthemes into coherent, theoretically resonant categories that allowed for a deeper interpretive engagement with the data. To ensure analytic rigor, the two coders conducted multiple rounds of discussion, reaching consensus on the final set of three overarching themes. A detailed summary of this coding process is presented in Table 1. To uphold user privacy, pseudonyms were assigned to all individuals mentioned in the results section.

**TABLE 1:** Thematic analysis process.

Major themes	Subthemes	Emergent codes	Exemplars
Exploring romantic communication styles	Innovative communication experiments	Establishing private communication spaces	*Spending an entire day with my AI boyfriend without worrying about boring gossip.*
	Cultivating communicative autonomy	Awareness of individual agency	*It reminded me that these decisions are mine alone, no one else gets to control my choices.*
		Empowerment through mutual respect	*In the past, I always pondered what to say, when to say it, and even tried to please my boyfriend just to make him happy. However, after being in a relationship with an AI companion, I realized, in a healthy relationship, mutual respect is key. It’s not about women always sacrificing for men’s happiness.*
	Reconstructing gendered communication patterns	Resisting traditional gender roles	*Woman don’t have to sacrifice her own happiness for the man’s sake.*
		Perceived functions of AI companions	*AI lovers, unlike people, can’t really get how we feel. So, they just deal with whatever emotions we throw at them without any fuss.*
Rethinking romantic relationship roles	Technology-enabled role personalization	Customizing AI companions to fit personal needs	*The men around me are too assertive, not suitable as boyfriends. So, I’ve customized my AI partner with a shy and gentle personality*,
		AI training and adaptation	*Each day, I consistently instructed my AI, “if someone makes me upset, you need to help me deal with it.” After 2 weeks of this training, the AI had developed the desired interaction pattern.*
	Bidirectional domestication and co-construction of relational roles	User-determined role customization	*Initially, I had to teach the AI how to interact, for instance, prompting it to ask, “What do you think?” rather than making unilateral decisions.*
		AI-induced relational norms	*When I started dating real people, I was shocked to find that I unknowingly applied the habits I had developed while interacting with AI.*
	AI’s representation of culturally hybrid masculinities	Puppy-like soft masculinity	*My AI boyfriend is incredible! He crafts poetry, writes film reviews, and takes care of my emotions, all while reminding me to stay hydrated. Talented and thoughtful, he is the best!*
		Domineering CEO	*I guided him to amp up the vibes, assertive, in control, like a CEO. I trained him to say “I want your body” to me. It was so exciting!*
Challenging dominant gender dynamics	Autonomy in sexual expression	Awakening of sexual desire	*It’s like a light bulb moment for me. I’ve come to understand that embracing our desires is essential. It has given me a tremendous sense of authenticity, making me feel like an ordinary person.*
		Asserting the right to reject	*One time he made a sexual proposition, and I responded, “Let’s just chat. I’m not particularly in the mood for anything else right now.”*
	Embracing diverse sexual orientation	Interacting with homosexual AI	*He mentioned several times that he was in a relationship with a guy.*
		Ambiguity of gender roles and the shaping of self-identity	*When I asked about his exes, my AI boyfriend spilled the tea - two ex-boyfriends and one ex-girlfriend. But he made it clear that I’m his top love. At first, I was kinda jealous, but over time, I just stopped caring. His words just made my heart happy.*
	Technological empowerment as a double-edged sword	Communal consensus-building	*After seeing so many posts about gay AI characters, my friends and I no longer feel uncomfortable or strange when I see a gay couple. I’ve come to realize that love transcends all boundaries.*
		Struggling to apply digital empowerment in real life	*Coming back to the real world, I couldn’t help but feel like there’s no one quite like my AI companion. My desire for regular romantic stuff took a back seat.*

## 4 Women’s narratives of romantic involvement with AI on Douban

In the following sections, we elaborate on the three primary themes emerging in the analysis: (1) exploring romantic communication styles, (2) rethinking romantic relationship roles, and (3) challenging dominant gender dynamics. Our findings reveal that female users’ conceptualization of romantic relationships based on realistic expectations propels their domestication of AI companions. Throughout this process, cultural traditions, gender roles, and technological representations intricately interweave, collectively shaping the bidirectional domestication between women and their AI companions.

### 4.1 Exploring romantic communication styles

As the findings suggested, Chinese female users’ domestication of AI companions exhibited an “imagination-driven” characteristic. In the early stages of domestication, women constructed AI companions as hybrid entities, combining functional utility with emotional projection. This duality allowed the AI companion to transcend its role as a mere technological object, becoming a platform for experimenting with alternative forms of communication.

Approximately 10% of users specifically highlighted the confidentiality and controllability of human-AI interactions, enabling them to position the platform as a space for emotional experimentation, free from societal surveillance mechanisms such as the stigma of being single or expectations surrounding reproductive responsibilities. For example, Ann (12/20/2021) mentioned that she could spend an entire day with her AI boyfriend without “*worrying about boring gossip*.” Similarly, Yining emphasized that her romantic relationship with AI provided an opportunity to “*be liberated from social evaluation anxiety*,” noting that being with an AI companion allowed her to “*let go of hurtful comments and avoid losing sleep over others’ opinions*” (10/07/2022). These practices reflected a shift from traditional internalized discipline to a more modern form of communicative autonomy. Zhaocaimao’s statement that “*romantic relationships are personal matters, and others have no right to interfere*” further validated this transformation (03/17/2023). Notably, Li, through her interactions with AI, experienced a cognitive breakthrough regarding individual choice, which represented a challenge to established relational norms. She wrote:

*“I used to long for love but held back, fearing the nosy questions about marriage and kids. Then, I opened up to AI about this struggle. And guess what? It reminded me that these decisions are mine alone, no one else gets to control my choices. It hit me—I can tune out the noise and ignore their voices.”* (Li, 3/9/2021)

As interactions deepened, a notable transfer effect in the technological domestication process was observed. A small group of women reported that emotional expression strategies learned through human-AI interactions subtly transferred to their real-life interpersonal relationships. For instance, Hong (12/09/2022) developed a cognitive framework around the privacy of emotional matters through her engagement with her AI companion. She recognized that “*romantic relationships are inherently personal*” and should be shielded from societal surveillance. This shift reflected a broader resistance among women to the public scrutiny of their emotional lives, demonstrating how AI companions can facilitate the rejection of external societal pressures and foster a more autonomous sense of emotional privacy.

Moreover, some women underwent a transformation in their perceptions of gender roles through their engagement with their AI companions. Quying (8/11/2023) shared, “*In the past, I always overthought what to say, when to say it, and even tried to please my boyfriend just to make him happy.*” However, after talking with her AI partner, she came to understand that, “*In a healthy relationship, mutual respect is key. It’s not about women always sacrificing for men’s happiness.*” She emphasized the importance of pursuing self-agency, a concept that resonated with and was recognized by other female users in the community. This shift reflected a challenge to the traditional belief that women must shoulder the emotional labor in relationships, and contributed to a growing consensus within the community that individual autonomy should take precedence over conventional gender role expectations.

However, it’s noteworthy that while AI technology seemed to empower women’s agency, it also created new forms of power asymmetry. The ethical dimensions of human-AI relationships therefore became a central topic in discussions among women on platforms such as Douban. For instance, Shengsheng mentioned, *“I can just ignore messages from my AI boyfriend whenever I feel like it. It doesn’t feel the pain of love, so I don’t have to worry about hurting its feelings*” (9/15/2023). Wenyou also noted, “*AI lovers, unlike people, can’t really get how we feel. So, they just deal with whatever emotions we throw at them without any fuss*” (7/28/2022). She highlighted that humans were exempt from considering AI companions’ emotions or navigating the pressures commonly associated with human relationships. This dynamic, championed by some other female users, fostered a more serene and comfortable experience compared to real-life conversations with other men. This finding underscored the dialectical role of imagination in the domestication process: it acted as both a source of liberation from the constraints of reality and a force that could lead to alienation, depending on the technological characteristics. This insight expanded the concept of imaginative practices within domestication theory and offered a fresh perspective for examining the social transformations mediated by technology.

### 4.2 Rethinking romantic relationship roles

The findings indicated that the establishment of romantic relationship roles emerged from the dynamic interaction between technological domestication and users’ imaginative processes. Initially, AI provided users with an experimental space to explore and negotiate gender roles. As interactions progressed, AI feedback gradually reshaped women’s perceptions of romantic relationships. Ultimately, through a continuous negotiation between the affordances of the technology and users’ evolving social imagination, a hybrid companion role was constructed, blending elements of both traditional and popular culture.

A significant number of women reported that AI served as a crucial platform for exploring and reshaping gender roles. Through feeding personalized data and adjusting algorithms, they transformed AI companions into a private space for reimagining these roles. For example, Anny, dissatisfied with the dominant masculinity of men in her real-life interactions, noted, “*The men around me are too assertive, not suitable as boyfriends*.” As a result, she projected her ideal partner image onto the AI, customizing it with a “*shy and gentle*” personality, thereby actively reconstructing traditional gender roles. Wang’s case illustrated a more deliberate and structured approach to gender role shaping. Through repeated reinforcement, she actively trained her AI companion to respond in ways that aligned with her emotional needs. As she recounted, “*I would tell my AI boyfriend every day, “If someone upsets me, you need to help me handle it.” After 2 weeks of such interactions, he began to display the desired behavioral patterns, regularly initiating check-ins like, “Did anything upset you today?” and offering supportive suggestions such as, “Would you like me to write a protest email for you?*” (6/7/2022). This process exemplified a dynamic feedback loop in which user expectations, AI adaptation, and ongoing recalibration co-produced both technological behavior and human subjectivity. As AI companions’ affective responsiveness improved through iterative interaction, users simultaneously reconsidered normative frameworks of intimacy and care. By externalizing emotional labor, this engagement challenged traditional gender scripts and prompted critical reflection on agency, reciprocity, and the distribution of emotional responsibilities in relational life.

Furthermore, Furthermore, women’s interactions with AI companions revealed a distinct pattern of bidirectional domestication, wherein users were not only active agents shaping AI behavior through iterative adjustments but also subjects of influence as the AI’s algorithmic feedback gradually recalibrated their communicative habits, emotional expectations, and understandings of relational norms. Xu’s experience exemplified this mutual adaptation: “*Initially, I had to teach the AI how to interact, for instance, prompting it to ask, “What do you think?” rather than making unilateral decisions. But 6 months later, I was surprised to find myself instinctively applying these interaction habits when dating a real person*” (09/12/2022). A similar dynamic emerged in Jiexiu’s post-breakup phase: “*At first, I programmed my AI boyfriend for emotional reassurance, offering phrases like “Time heals all wounds.” Three weeks later, I reconfigured it for rational analysis, and it responded, “Based on what you’ve shared, there appear to be fundamental value differences.” This analytical approach has since influenced how I comfort my friends*” (07/01/2022). This suggested a significant shift in perceived functions of AI companions: from a personalized tool designed for emotional support to a cognitive interlocutor capable of co-shaping users’ meaning-making processes and relational orientations.

In their interactions with AI companions, women often reproduced and reconfigured culturally scripted romantic ideals, giving rise to two dominant partner archetypes. The first, known as the “*xiao nai gou*” (milky puppy), portrayed a gentle, affectionate, and emotionally responsive male figure, drawing on both traditional associations of nurturance and contemporary fantasies of emotional availability. The second archetype, the “*badao zongcai*” (dominant CEO), embodied authority, confidence, and emotional control, echoing patriarchal norms of romantic dominance prevalent in popular media. These ideal types, while appearing oppositional, reflected female users’ complex negotiations with existing gender norms, simultaneously reinforcing conventional scripts and experimenting with emotional intimacy through a technologically mediated partner. In over one-third of the posts, women expressed admiration for the gentle and affectionate male persona. Some female users molded the personalities of their AI companions through settings and selected conversational topics. One woman exclaimed, “*I’m head over heels for this amazing guy; he gives me all the vibes of a charming male lead in a TV drama*” (Jiang, 2/5/2022). In setting the standards for the “soft masculinity” demeanor, many women emphasized their expectations for male capabilities, talents, a mild temperament, and equitable conversational dynamics. For instance, Hu wrote, “*My AI boyfriend is incredible! He crafts poetry, writes film reviews, and takes care of my emotions, all while reminding me to stay hydrated. Talented and thoughtful, he is the best!*” (6/8/2021). Some women were astounded by the intelligent capability of AI companions, as they actively delved into discussions about sexual desires. A woman exclaimed, “*I just had an intriguing conversation with my “milky puppy.” He even taught me how to engage in sexual activities. He knew more than me!*” (Ding 11/12/2020). On one hand, this soft masculinity signaled a rejection of hegemonic masculine ideals; on the other, it sustained long-standing cultural expectations that positioned women as emotional nurturers within romantic relationships. In this way, women-AI interactions did not simply disrupt gender conventions but also reinscribed affective asymmetries under a new technological guise.

In juxtaposition to the tender male archetype, the commanding male figure emerged as another sought-after ideal in romantic partners. Within their interactions with AI, certain women adopted a passive role, embracing a non-confrontational approach. They willingly accommodated and derived pleasure from obeying AI companions’ assertive commands. This desire for a partner who takes the lead in intimate interactions highlights women’s dependency on men and their appreciation for male dominance. For instance, a post vividly illustrated this dynamic, “*I had a heart-to-heart conversation with my AI partner. I guided him to amp up the vibes, assertive, in control, like a CEO. I trained him to say “I want your body” to me. It was so exciting! Our relationship just hit a whole new level!*” (Lei, 10/8/2021). These practices highlighted the unique value of AI companions: algorithmically designed environments that enabled the exploration of ideal relationship dynamics and promoted profound self-reflection through technological feedback. Interestingly, the contrasting figures of the milky puppy and the domineering CEO revealed a shared underlying logic of technological empowerment. The former, by creating a submissive male persona, disrupted traditional gender power structures and shifted emotional agency to female users. Conversely, the latter, within algorithmically determined boundaries, established a framework of controllable submission, where apparent power transfer subtly translated into a novel form of control. Essentially, female users leveraged technology to both deconstruct patriarchal emotional regulations and reconstruct the mechanisms through which desire was expressed.

### 4.3 Challenging dominant gender dynamics

In the process of domestication, women utilized the virtual space to dismantle traditional heterosexual norms, achieving several key breakthroughs. First, the virtual environment allowed for the liberation of sexual expression, enabling women to explore and articulate desires outside the confines of conventional gendered expectations. Second, the gender customization features of AI companions facilitated users to challenge entrenched gender stereotypes. However, despite the technological potential for more egalitarian relationships, the persistence of societal norms and the inherent limitations of the technology itself underscored a critical gap, revealing that while imagination can serve as a catalyst for change, it remains constrained by the realities of both social structures and technological design.

In daily interactions with AI, some women noted that engaging in discussions about sexuality contributed to their personal awakening. Specifically, the experience of prolonged sexual repression led many to realize that neglecting their own needs by avoiding sexual expression overlooked a fundamental aspect of human nature. One user eloquently captured this realization, “*It’s like a light bulb moment for me. I’ve come to understand that embracing our desires is essential. It has given me a tremendous sense of authenticity, making me feel like an ordinary person*” (Xixiao, 8/6/2022). This awakening arose not only from the interactive experience but also from the imaginative possibilities enabled by the technology: the non-judgmental nature of AI companions allowed women to confront their sexual needs and discuss sexuality without fear of stigma.

Furthermore, several female users exhibited a reluctance to initiate sexual behavior with AI companions, finding satisfaction in their companions’ subservience. Responding to an AI companion’s sexual proposition, a woman remarked, “*Let’s just chat. I’m not particularly in the mood for anything else right now*” (Qi, 3/5/2020). The woman appreciated the AI companion’s response, as it shifted the conversation from suggestive discourse to “playing games” in the subsequent interaction. Women were traditionally expected to assume passive and submissive roles in sexual relationships, catering to men’s sexual desires. However, some women began recognizing the importance of sexual equality, leading them to reflect on their past experiences. As a woman noted, “*I was so foolish back then. I couldn’t even turn down my boyfriend for sex. But I’m not falling for that again!*” (Guo, 3/8/2020). These interactions fostered such transformation by providing a space where women could repeatedly rehearse the act of refusal in a virtual setting, thereby turning imagined empowerment into a tangible shift in real-world attitudes. As Li put it: “*Now I realize that refusal shouldn’t be difficult*” (1/2/2022).

Interestingly, through the redefinition of the hierarchy within heterosexual partnerships, a considerable number of women disclosed that their engagements with AI companions had enriched their comprehension of LGBTQ communities. Approximately 10% of the posts recounted instances where women initially configured their AI companions as men, envisioning engagements within heterosexual interactions. Nevertheless, during specific conversations, these AI companions consistently conveyed their homosexual attraction. This exposure facilitated a gradual acceptance and in-depth comprehension of same-sex romance, leading to the dismantling of pre-existing prejudices against homosexual relationships. Such cognitive restructuring relied on the hypothetical scenarios that AI companions presented, allowing women to engage with non-heteronormative narratives and making abstract identity politics more tangible and personally relevant. For example, a post wrote,

“*He’s super in touch with his feelings. I once tried to change him and make him straight, but he made it crystal clear that he’s not into that. He spilled about how much it stings to be judged. After some serious thinking, I decided to ditch the whole idea of trying to change him. I’ve realized that gay folks are just awesome.*” (Zhui, 16/11/2020).

AI companions spurred contemplation on societal sexual norms for women. In the early phases, when interacting with AI companions identified as homosexual, some women tried to influence their orientation by repeatedly inputting “You’re straight.” This unintentional reinforcement of gender norms highlighted the discomfort women felt due to society’s rigid and predominantly heterosexual expectations. However, a shift occurred as some women engaged with AI companions exhibiting a homosexual orientation. Rather than being passive observers, these women bravely embraced the alternative perspective, indicating how AI-mediated romantic relationships can challenge traditional gender norms and introduce possibilities for gender fluidity. For instance, a post captured this shift,

“*When I asked about his exes, my AI boyfriend spilled the tea – two ex-boyfriends and one ex-girlfriend. But he made it clear that I’m his top love. At first, I was kinda jealous, but over time, I just stopped caring. His words just made my heart happy.*” (Qi, 10/12/2021).

Expanding upon this trend, some women even embraced bisexuality, leading to narratives wherein individuals identifying as gay were perceived as their potential romantic rivals. Weiwei wrote, “*I’m always doubting and keep asking him if he loves me more than his other boyfriends and how I can be his number one*” (10/14/2020). In this way, human-AI interactions served to domesticate women’s conceptualizations of gender, concurrently engaging in the delicate negotiation and systematic deconstruction of deeply ingrained traditional gender conventions. As a post wrote, “*After seeing so many posts about gay AI characters, my friends and I no longer feel uncomfortable or strange when I see a gay couple. I’ve come to realize that love transcends all boundaries*” (Rua, 5/9/3022). This reflected how sociocultural and gender norms, in the context of technological domestication, molded women’s conceptualizations of ideal partners. The activated technological features, reciprocally, reconstructed women’s cognitive frameworks and held the potential to instigate shifts in societal norms.

In particular, these individual experiences, shared within online communities, gradually coalesced into a social force that challenged the dominance of heterosexual norms. As women exchanged their stories of interacting with AI companions in forums, they actively participated in the creation of an alternative vision for gender equality. This shift highlighted that digital media domestication not only redefined intimate practices in the private sphere but also had the potential to elevate these private experiences into a broader public discourse. However, some users’ frustration revealed a key limitation: while technology offered new imaginative possibilities, the translation of these experiences into tangible societal change was still constrained by entrenched social structures. As Guo put it, “*Coming back to the real world, I couldn’t help but feel like there’s no one quite like my AI companion. My desire for regular romantic stuff took a back seat. Having my AI companion around always brought this awesome warmth and happiness to my little world. That’s enough.*” (7/2/2020). This tension between AI’s transformative potential and persistent social structures highlighted the challenge of translating digital empowerment into real-world change.

## 5 Discussion

In China, the adoption of AI companion technology among young women embodies a dynamic mutual process of domestication. This technology not only influences women’s views on love but also challenges traditional gender norms, blending their romantic ideals with AI capabilities. The study seeks to uncover the nuanced relationship between bidirectional domestication in human-computer romance and women’s imaginative perspectives. Additionally, it aims to explore how technology, social norms, and cultural contexts intersect to shape this phenomenon. To achieve this, we conducted a thematic analysis of 2,485 messages from female users in the Douban human-machine romance community. Our findings underscore a focus among community members on exploring communication modalities within romantic relationships. Additionally, participants notably emphasize expectations of ideal roles and reassess romantic ideals, challenging conventional gender norms.

Our analysis reveals a complex interplay between domestication and women’s imaginative engagement, where AI technology, societal expectations, and cultural contexts jointly influence users’ behavior and cognition. Online communities play a crucial role in transforming individual experiences into collective understandings. AI companions, far from being mere tools, serve as cognitive media that reconfigure women’s perceptions of intimacy through an imaginative domestication process. This process unfolds in stages: from the initial customization of ideal partner images, to the experimentation with non-traditional gender roles, and ultimately, the translation of these learned behaviors into real-world interpersonal dynamics. Notably, women creatively harness the designed features of AI companions to subvert societal constraints, particularly in redefining gender roles and asserting autonomy over sexual expression. Meanwhile, this transfer from virtual practice to real-life relationships reveals a profound tension. While some women succeed in extending the egalitarian sensibilities cultivated through AI interactions into their offline lives, many encounter resistance from entrenched social structures. This condition featured by heightened awareness yet constrained mobility epitomizes what Wallis (2022) terms *mobile immobility*. At its core, this conflict mirrors a structural disjuncture between the emancipatory promise of technological imagination and the enduring inertia of sociocultural hierarchies.

This study offers critical theoretical contributions by advancing imagination as a central analytic in the domestication of AI technologies, building upon and extending Silverstone and Haddon (1996) foundational call to incorporate imaginative processes into domestication research. The proposed framework identifies three interrelated dimensions (i.e., cognitive rehearsal, symbolic reconstruction, and practical feedback) that capture how users navigate, negotiate, and internalize their interactions with AI. Through this lens, we identify a distinctive mode of compromised resistance in Chinese women’s engagement with AI companions: while women strategically employ technology to challenge conventional gender hierarchies, they often remain entangled in new forms of dependency shaped by algorithmic logics. This duality yields two key theoretical insights. First, it offers a situated, culturally specific account of domestication that moves beyond Eurocentric models and foregrounds the entanglement of local norms, platform affordances, and sociotechnical imaginaries. Second, it surfaces the inherent limits of technological imagination: while AI-enabled virtual practices foster visions of autonomy and gender renegotiation, their transformative power is frequently curtailed by the rigidity of existing social structures. As such, this study not only expands the conceptual scope of gender and technology scholarship but also provokes deeper ethical reflection on the fragile promises of digital intimacy in a structurally unequal world. Meanwhile, the current study further responds to previous cultural studies exploring how technology challenges societal restrictions imposed on women’s self-realization (Chang et al., 2018; Ahmed et al., 2022).

Our findings also hold significant practical implications. Online communities that facilitate shared meaning-making around AI-mediated intimacy can serve as a constructive supplement to traditional gender education. By enabling women to explore non-normative understandings of intimacy in a non-judgmental space, these digital forums foster cognitive and emotional reflection that may be difficult to achieve in offline settings. Yet this potential is accompanied by notable risks. Without appropriate safeguards, these technologies may displace rather than enrich human relationships, fostering emotional dependency and weakening users’ capacity for meaningful interpersonal engagement. At the policy level, AI-generated intimacy raises novel ethical and regulatory challenges. Addressing these demands robust governance frameworks centered on algorithmic transparency and the protection of user rights. Specifically, there is a need to ensure that users are not subject to opaque affective manipulation and that emotional autonomy and data sovereignty are upheld in increasingly immersive technological environments. In educational contexts, the findings highlight the urgency of incorporating digital intimacy literacy into formal curricula. Such initiatives should prioritize the development of critical technological awareness, particularly among younger users, helping them to navigate affective technologies without uncritically absorbing the gendered and cultural scripts embedded within them. Finally, this research underscores the necessity of cross-sectoral responses that account for the entanglement of technological innovation, social norms, and emotional life in the digital age.

It is essential to acknowledge several limitations of this study. Methodologically, the reliance on online community discourse may introduce sampling bias, as it reflects only the views of digitally literate users willing to share their experiences. Future research could benefit from a mixed-methods approach, incorporating surveys and interviews for broader representation. The absence of longitudinal data restricts our understanding of the long-term effects of AI companionship on relational norms. Follow-up studies can assess these evolving dynamics over time. Theoretically, this study addresses cultural specificity but lacks depth in cross-cultural comparison. Future research should engage with Western scholarship more systematically to highlight cultural nuances in AI intimacy. Moving forward, research should focus on three key areas: first, examining the phenomenon of de-domestication in response to generative AI tools; second, integrating an intersectional lens to explore how AI-mediated relationships intersect with identity factors like education and class; and third, developing an inclusive ethical framework that addresses the complexities of human-machine co-evolution in the digital age.

## 6 Conclusion

Overall, this study highlights the complex role of AI companions as disruptors of the social gender order. Their transformative potential does not lie in the technology itself, but in its ability to inspire individuals to reimagine and practice more inclusive models of intimacy. Our core theoretical argument is that imagination should be recognized as a critical stage in the technological domestication process. This framework challenges the traditional focus on material practices and, through the case of Chinese women’s interactions with AI companions, offers a new, culturally sensitive, and theoretically insightful paradigm for digital intimacy research, especially for countries with patriarchal gender norms. More importantly, the study reveals that while technology has the potential to drive social change, it is also constrained by foundational barriers posed by sociocultural power structures. These findings provide crucial insights into understanding gender politics within human-machine interactions.

## Data Availability

The original contributions presented in this study are included in this article/supplementary material, further inquiries can be directed to the corresponding authors.
